# Investigating the Role of Prolactin as a Potential Biomarker of Stress in Castrated Male Domestic Dogs

**DOI:** 10.3390/ani9090676

**Published:** 2019-09-12

**Authors:** Jara Gutiérrez, Angelo Gazzano, Federica Pirrone, Claudio Sighieri, Chiara Mariti

**Affiliations:** 1 Department of Veterinary Sciences, University of Pisa, 56124 Pisa, Italy; angelo.gazzano@unipi.it (A.G.); claudio.sighieri@unipi.it (C.S.); chiara.mariti@unipi.it (C.M.); 2 Department of Veterinary Sciences, University of Milano, 26900 Milan, Italy; federica.pirrone@unimi.it

**Keywords:** behaviour, cortisol, dog, male, prolactin, shelter, Spanish Greyhound, stress

## Abstract

**Simple Summary:**

Although cortisol is usually considered the main reference for the assessment of stress, in some animal species it has been shown that prolactin can be used as a biomarker of both acute and chronic stress. Behavioural parameters can also be used to assess the state of welfare and stress. This study was aimed at evaluating the possible relationship between serum prolactin, serum cortisol and behavioural signs of stress in domestic dogs. To reduce the possible influence of some factors, the study was performed on a homogeneous sample formed by 40 castrated male Spanish Greyhound dogs housed in a dog shelter. The weak negative correlation found between serum cortisol and prolactin values agrees with results obtained in other studies, indicating that prolactin response might be an alternative to cortisol response.

**Abstract:**

Prolactin has been recently regarded as a potential biomarker of both acute and chronic stress in several species. Since only few studies until now have focussed on domestic dogs, this study was aimed at evaluating whether prolactin, cortisol and stress behaviour correlated with each other in sheltered dogs. Both cortisol and prolactin analysis were performed in serum samples through a hormone-specific ELISA kit. For each dog, a stress score was calculated by summing the number of occurrences of stress-related behaviours. The presence/absence of fear during the time spent in the collection room was also scored for each individual. Results revealed a weak negative correlation between cortisol and prolactin levels. Neither of the hormones was correlated with the stress score, nor did their values seem to be influenced by showing fear in the collection room. The weak negative correlation found between cortisol and prolactin values agrees with results obtained in other studies, indicating that prolactin response might be an alternative to cortisol response. This, together with the high serum prolactin levels compared to those reported by other authors for healthy domestic dogs, may indicate that prolactin might be a good biomarker of chronic stress, and although further studies are needed to better understand the potential role of prolactin in the evaluation of canine welfare.

## 1. Introduction

Although prolactin hormone is known for its function in the stimulation of the growth of the mammary gland and the lactation processes, it has more than 300 different biological activities, homeostatic roles and physiological functions in the organism, e.g., electrolyte balance, luteal function, regulation of the immune system, osmoregulation, angiogenesis, and maintenance of the inter-oestrous interval [1]. In fact, the important genetic role of the prolactin receptor (PRL-R) in energy balance and metabolic adaptation has been recently evidenced in rodents, proving that prolactin has essential roles for the metabolism of glucose, insulin and lipids, as well as in promoting a positive energy balance [2]. Furthermore, prolactin has been found to be an index of acute stress in some non-human animal species, such as rats [3,4,5], domestic ruminants [6], donkeys [7], cattle [8], and sheep [9]; and different specific functions of prolactin during the stress response have also been demonstrated [2], such as mediation in the epidermal adaptation to environmental stress in fish [10].

It has been recently found that prolactin signalling in tuberoinfundibular dopamine (TIDA) neurons was reduced in mice exposed to acute stress, with a consequent potential decline in their inhibitory influence on prolactin secretion [11]. This suggests that prolactin secreted in response to acute stress may activate prolactin receptors in certain tissues involved in physiological adaptation to stress [11]. In humans, prolactin is stimulated by suckling, perception of visual, acoustic and olfactory stimuli, as well as by stress [12], including psychological and psychosocial stresses [13], and experimental stress-related conditions, such as hypoglycaemia [14], surgery [14], parachute jumping in military recruits [15], and compulsory swimming in non-swimmers [16]. In fact, cortisol, prolactin and day-to-day changes in anxiety indexes measured by questionnaires were found to be significantly correlated [17].

However, the scientific literature reports divergent results with both unchanged [18,19] and decreased prolactin levels [20,21,22] in response to stress, questioning the belief that stress stimulates prolactin secretion [23]. In addition, while cortisol levels were found to correlate with anxiety levels the day before a surgical intervention, no significant correlation between prolactin and anxiety was found [24].

As for the domestic dog (*Canis familiaris*), to our knowledge the potential link between stress and prolactin levels has been scarcely investigated. One study assessing surgery-related stress in dogs found that cortisol increased and prolactin decreased in the post-surgery period compared to basal levels [25], the latter being in contrast with increased post-operative prolactin response found in humans after surgery [26]. The authors suggested that this contrast may be showing that different species can exert differences in the activation of prolactin feedback regulatory systems [25].

A few studies have reported that dogs with generalized anxiety had hyperprolactinemia, but dogs with phobias or mild anxiety did not [27,28]. Prolactin blood levels were positively correlated with the score obtained on an individual scale for quantifying the presence of anxiety-related behaviours through a physical and behavioural evaluation [28]. In fact, dogs suffering from different kinds of generalized anxiety showed a significant decrease in dopamine blood levels [29], which can justify measuring prolactin, since its secretion is mainly controlled by dopaminergic neurones [28]. However, the involvement of prolactin in emotional responses in dogs seems to be more complex, as its circulating concentrations have been shown to increase also during positive interaction with humans [30].

Cortisol is commonly regarded as the “stress hormone”; cortisol and its metabolites have in fact been quantified in various sample matrices such as blood, saliva, urine and faeces [31], and more recently in hair [32,33,34], as an endocrine response to stress. Cortisol levels have been shown to increase in adverse conditions such as isolation, restriction of movement, regrouping or transport [35]. In fact, cortisol is secreted following the activation of the hypothalamic–pituitary–adrenal (HPA) axis, one of the major stress response systems [36]. Nonetheless, increased HPA activity is not stress-specific, since it can also be caused by metabolic processes, positive affective states, maternal behaviour and physical activity [37,38,39,40]. For this reason, some authors suggest that an increase in cortisol levels should be regarded as an indicator of arousal [37] rather than of stress.

The reliability of cortisol in the assessment of stress has further been questioned due to many factors that can affect the interpretation of cortisol levels such as individual variability in the response to a stressful exposure [41,42], a high inter-individual variability in baseline cortisol levels between dogs [43], distress for blood sampling [44], and possible variations due to the circadian rhythm [44], the latter being not clearly identified in domestic dogs: in fact, no circadian rhythm for cortisol has been reported for laboratory [45] and working dogs [46,47] for intervals of 24–28 h. All these factors suggest caution when comparing cortisol levels in groups of different individual dogs, and when assessing long-term cortisol secretion from fluids such as blood, urine and saliva [44]. The evaluation of cortisol levels can be further complicated by changes with respect to the duration of stress. Whereas in the presence of an acute stressor serum, cortisol rapidly increases and then returns to the basal levels, in a chronic stress situation, a prolonged exposure to the stressor can lead to the suppression and deregulation of the HPA axis [48], which generally does not mean a normalization of circulating cortisol levels, and thus, they are a very useful measure of chronic stress [49].In recent years, hair cortisol analysis has also been supported as a reliable reflection of long-term cortisol secretion [50,51].

Cortisol and prolactin seem to both be involved in the stress response and their levels are likely to be somehow associated. For instance, in vitro experiments showed higher basal levels of corticosterone in hyperprolactinemic rats than in normal and hypoprolactinemic ones; and prolactin was found to exert a stimulating effect on ACTH-induced corticosterone secretion in acute restraint stress [52]. Nevertheless, the response of both prolactin and cortisol hormones to a stressful situation may be divergent, and some authors suggest that prolactin release can act as an alternative form of the cortisol response to stress [22].

In dogs, the evaluation of stress is often a combination of physiological and behavioural parameters, the latter being usually considered a reliable indicator [53,54]. For instance, after the induction of chronic stress through a model of social and spatial restriction, dogs living in small indoor kennels showed significantly lower postures [55], as well as an increase in cortisol levels [56] compared to those living in enriched spacious outdoors housing in groups. When exposed to short-kennelling environment, dogs were generally more active [36,57], and their cortisol:creatinine ratios (C/Cr) were significantly higher compared to when they were in home environment [36].

The aim of this opportunistic study was to evaluate whether there is a correlation between serum cortisol and prolactin concentrations and between them and behavioural indicators of stress and fear in domestic dogs. To reduce the impact of possible affecting factors such as sex, neutering state, breed and housing condition, this study was carried out on a homogeneous sample of castrated male Spanish Greyhound dogs housed for more than 155 days in a dog shelter. This breed has a complex social context in the south of Spain, where this study was performed, with reports of abuse and neglect being frequent for them. Therefore, they could have experienced previous common adverse experiences prior to being rescued, which could reinforce the homogeneity of the sample.

The hypothesis of this study was that, as previously suggested by other authors, prolactin could be used as a biomarker of stress in domestic dogs and, for this reason, that it was correlated with other measures related to stress, such as serum cortisol, the presence of fear or the frequency of stress-related behaviours in dogs. Prolactin secretion from the pituitary gland is used as a marker for lactotropic axis activation [58], it has been found to be an index of acute stress in some species, and have specific functions during the stress response [2]. However, due to the controversial findings in the literature, we did not make any predictions about the possible increase or decrease of prolactin levels in a stressful condition.

This study includes some elements of novelty such as being, to our knowledge, the first including multiple measures of stress, both hormonal and behavioural (prolactin, cortisol and behaviours related to stress and fear), investigating their possible correlation, and involving such a homogeneous sample of domestic dogs, with all belonging to one single breed, being castrated males, and living in the same environment.

## 2. Materials and Methods

The procedure was communicated to the Ethics Committee of the University of Pisa, Italy and it received a favourable opinion with Decision N.09/2018.

### 2.1. Subjects and Place

Samples were collected between 3^rd^ September and 26^th^ November 2018 from 40 sheltered castrated adult male Spanish Greyhound dogs (mean age ± standard deviation = 46.5 ±20.8 months; min. = 19; max. = 112 months). Dogs were housed at the Fundación Benjamin Mehnert (Seville, Spain).

Dogs that entered the shelter were either wandering alone, probably after having been abandoned, occasionally hit by a car, or coming directly from hunter discards or reports of abuse and mistreatment. All of them were hosted in the shelter for a period longer than 155 days (270.9 ± 115.6 days), and had a healthy condition, regularly checked by a veterinarian.

The shelter included a covered space of 2000 m^2^ divided into 2 rows and 3 main corridors, with a total of 60 boxes, each of which was provided with 1-3 plastic beds (88 × 58 × 28 cm), depending on the number of dogs sharing the box. Apart from the feeder and the water bucket, there were no other objects such as toys in the boxes. Dogs went out to the outer courtyards in small groups twice a day.

Dogs were usually living in a group of 3–4 dogs (92.5% of cases), all Spanish Greyhounds, sharing the same box for days or weeks; in several cases (n = 3), five dogs were housed together. The shelter staff organized dogs in groups that ensured the greatest possible social stability, thus partially counterbalancing the risk of stress due to overcrowding or conflicts.

### 2.2. Video Analysis

A 10-min video was recorded of each dog using a video camera (JVC GZ-MG130E, Yokohama, Japan) located about one meter away from the box fence (the maximum possible distance to cover the whole box) and at a stable height of 100 cm. Videos were recorded between 8:30 a.m. and 3:00 p.m. To facilitate the recognition of each dog in the videos, their most peculiar physical characteristics were registered, and a picture was taken. 

Videos were analysed using BORIS (Behavioral Observation Research Interactive Software, University of Turin, Turin, Italy) [59]. A list of 18 possible signs of stress in dogs was created [54,60,61,62,63], and for each of them the number of displays (observations) was recorded. A stress score was calculated for each dog, by summing up the total number of times each of the following stress-related behaviour was displayed: yawning, shaking, paw lifting, tongue out, eliminating, growling, turn head, tuck tail, cowering, trembling, circling, pacing, hiding, panting, salivation, howling, whining and behaviour against the fence (any behaviour in which the dog physically contacted the fence, such as jumping on the fence or standing up against it).

Videos were analysed by an observer having experience with video analysis of dog behaviour. To measure observational accuracy, a second observer, also having previous experience in behavioural analysis of videos, analysed 3 videos using the same software (BORIS), and inter-observer agreement was calculated.

### 2.3. Presence of Fear in the Collection Room

After recording the video, 2–3 dogs were contemporaneously led on the leash to a room within the shelter facility, from here called collection room. This movement required around two minutes. In the collection room, dogs were restrained using a leash tied to a table.

The presence of fear around the time of blood sampling was qualitatively scored (yes/no), as a result of a global consideration of the dog behaviour during the time spent in the collection room (20.6 ± 7.0 min). Fear was scored with a “yes” for those dogs expressing at least one of the behaviours related to fear [64], such as the tail tucked, trembling, cowering, and hiding behind objects or in a corner, avoidance of eye contact and approach, and freezing.

### 2.4. Serum Samples Collection and Storing

Blood (3–4 mL) was drawn from the cephalic vein in the collection room by a veterinarian, part of the shelter staff and familiar to all the dogs, avoiding stress for the dog as much as possible. Only the veterinarian was involved in both restraining the dog during the collection and blood withdrawing.

Based on previous literature about the times at which prolactin and cortisol reach their maximum values and decreased in blood after a stressful situation in humans [65,66], samples were collected about 10 min (ranging from 5 to 15 min) after entering the collection room. When the procedure (identification, blood sampling, and clinical examination) was completed for all the dogs in room, they were moved together to their box.

Tubes containing the blood were kept upright refrigerated (4 °C) in a padded box. After 50–90 min, blood was centrifuged at 3000 rpm for 20 min (using Nahita 2615 Auxilab SL, Beriain, Spain). Serum samples were stored in Eppendorf tubes kept vertically in the freezer (−18 °C) and maintained frozen until being analysed.

### 2.5. ELISA Kit

Prolactin and cortisol concentrations from canine serum were measured using an EIA (enzyme immunoassay) kit (Demeditec Diagnostics^®^, Kiel, Germany for prolactin; Diametra^®^, Segrate, Italy for cortisol), according to the manufacturer’s instructions.

### 2.6. Statistical Analysis

A Shapiro-Wilk test (*p* < 0.05) was applied to the three parameters (prolactin, cortisol, and stress score) to investigate the normality of data. None of them was found to have a normal distribution; therefore, further analyses were carried out using non-parametric statistics.

A comparison between dogs showing fear and dogs not showing fear in the collection room was made for each parameter, through a Mann-Whitney U-test (*p* < 0.05).

A possible correlation between serum prolactin levels (ng/mL), serum cortisol levels (ng/mL) and stress score was investigated using Spearman’s rank correlation coefficient (*p* < 0.05).

## 3. Results

Regarding the videos, the percentage of inter-observer agreement was excellent (92.1%).

Results of stress-related behaviours are reported in Table 1, showing the number and relative percentage of dogs displaying each of the 18 analysed behaviours, and the number of occurrences (times the behaviour was displayed). Tongue out behaviour occurred with the highest frequency (total frequency = 101) and tuck tail with the lowest (total frequency = 1). Four behaviours (growling, hiding, salivation and trembling) were not displayed for any of the dogs (Table 1).

**Figure 1:**
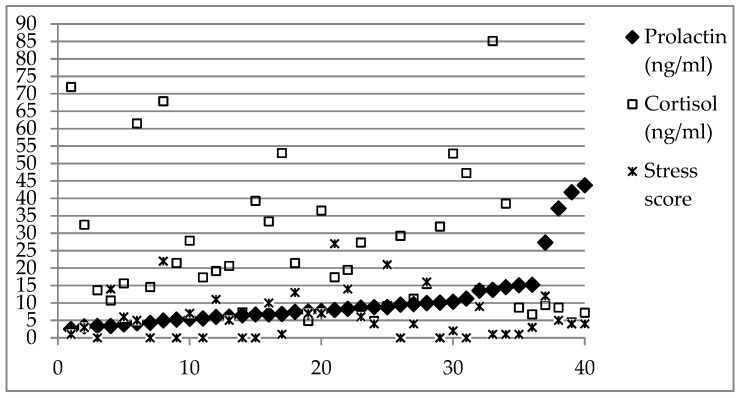
Results of serum prolactin, serum cortisol and stress score. Values for prolactin in serum (ng/mL), cortisol in serum (ng/mL) and stress score for each dog (n = 40) are shown. Data are organized according to increasing value of prolactin.

Thirteen dogs showed fear in the collection room and 27 did not. Results of the three parameters for individual dogs are reported in Figure 1.

**Figure 2:**
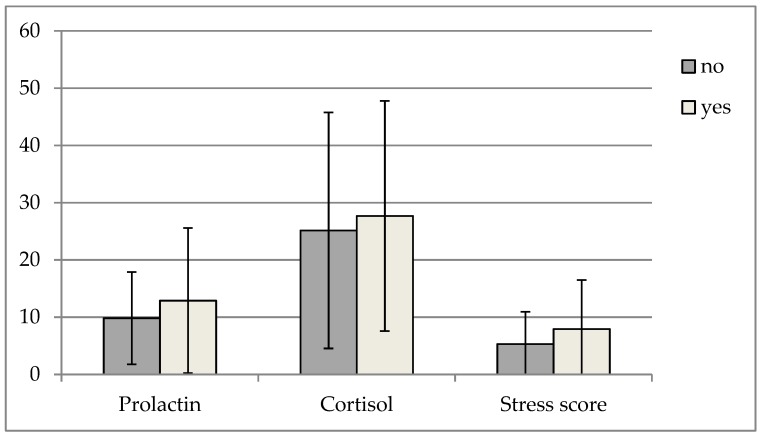
Results of serum prolactin, cortisol and stress score for dogs showing and not showing fearful in the collection room. Values for prolactin in serum (ng/mL), cortisol in serum (ng/mL) and stress score in dogs evaluated as showing (yes; n = 13) or not showing (no; n = 27) fearful behaviour in the collection room.

Prolactin values varied between 2.51 and 43.75 ng/mL, with a mean ± standard deviation of 10.82 ± 9.80 ng/mL. No difference in prolactin concentrations was observed when comparing dogs with and without fear in the collection room (*u* = 153.00; *p* = 0.516; see Figure 2).

Cortisol values ranged 4.43–85.14 ng/mL (25.97 ± 20.33 ng/mL). No difference was found in cortisol levels between fearful and non-fearful dogs in the collection room (*u* = 160.00; *p* = 0.654; see Figure 2).

Stress score values varied between 0 and 27 (6.15 ± 6.77). Stress scores did not differ between dogs with and without fear in the collection room (*u* = 143.00; *p* = 0.344; see Figure 2).

Serum prolactin levels and serum cortisol levels showed a weak negative correlation (Spearman Rho = −0.319; *p* = 0.045). The stress score did not show a correlation with either prolactin (Spearman Rho = 0.017; *p* = 0.918) or with cortisol levels (Spearman Rho = −0.155; *p* = 0.342).

## 4. Discussion

Scientific literature about prolactin normal ranges in dogs is relatively scarce. It is well documented, considering its role in lactation, that prolactin increases in pregnant and above all in lactating bitches, as well as in pseudopregnant bitches [67,68], with values ranging from 0 to around 40 ng/mL at 10 weeks after oestrus [67,69]. As for healthy male dogs, studies carried out on intact males have found different but similar ranges [70,71,72], which can be summarised as a normal range in intact male ranging from non-detectable to 6 ng/mL [71]. Some factors such as breed seem to be responsible for differences in prolactin secretion in intact male dogs [71,72], while age seems not to be influential [71]. The results of the current study refer to castrated males, to dogs housed in a shelter, and to a canine breed not previously involved in studies on prolactin, therefore their interpretation should be performed cautiously. However, data reported in Figure 1, as well as the mean value, show that most of the involved dogs had prolactin concentrations higher than the threshold indicated by Corrada et al. [71], and some dogs had values closer or higher than those reported by Pageat et al. [28] for anxious dogs treated with fluoxetine or selegiline (around 13 ng/mL).

Results on serum cortisol concentrations provided by this study showed a high individual variability, in accordance with Bennett and Hayssen [43]. The values obtained here are included within the wide range (5–100 ng/mL) for baseline cortisol measured by an ELISA kit in client-owned dogs’ serum [73]. However, other studies have reported a narrower range, varying according to the physiological state of dogs and the used analytical methods [74,75,76,77], making it difficult to compare and frame the values found in the current study. Generally speaking, the mean serum cortisol value obtained in this study was higher than the expected for healthy domestic dogs. The high cortisol values might be due to many factors, including a potential chronic stress condition lived by dogs in the shelter, with a consequent deregulation of HPA axis [48,56]. This will be discussed later.

Looking at the relationships between the investigated parameters, serum prolactin values were not correlated with stress score and they were weakly negatively correlated with serum cortisol levels. Several authors have reported that stressful situations can lead to higher circulating prolactin levels in various species [3,4,5,6,7,8,9,11,12,13,14,15,16], suggesting that prolactin might be a good biomarker of acute stress. Results of the current study seem not to support this hypothesis. However, other studies found that stress-linked cortisol changes did not affect prolactin levels [19,78,79] or have even reported a negative correlation between cortisol and prolactin levels [21,22,30,80,81]. Sobrinho et al. [22] suggested that this negative correlation might be due to the fact that prolactin release acts as an alternative form, rather than an extension of, the more common cortisol response, thus each hormone could be released in response to specific emotions [22].In fact, neural pathways responsible for cortisol and prolactin responses to stress are different in rodents [82], who often release prolactin in response to acute psychological stress [83]. Likewise, in sheltered dogs, whereas serum prolactin levels decreased in response to stress, no changes were observed in serum cortisol concentrations [58].

Prolactin has a direct influence on oxytocin secretion, and both hormones modulate the neuroendocrine acute stress response during maternal behaviour, having an anxiolytic effect in pregnant and lactating rats [84,85]. However, prolactin response to stress does not seem to be limited to females: in a study by Siracusa et al., there was no significant interaction between gender and change in serum prolactin concentrations among time points for dogs exposed to a synthetic, dog-appeasing pheromone both before and after surgery [58].

It must be noticed that the way in which individuals respond to stress and fear may depend on their individual history. Dogs of the current study have experienced situations of abuse, neglect and/or abandonment prior to entering the shelter, which can be all considered stressful situations. It can be hypothesized that their individual history may significantly have affected their way to respond to an acute stress situation. In fact, dopamine release has been shown to increase in response to acute stress in rats if they had previous exposure to chronic stress [86]. This was in agreement with the decrease found in prolactin concentrations in sheltered dogs with assumed chronic stress that were exposed to acute stress [25].

In the current study, prolactin concentrations did not correlate neither to stress score nor to fear behaviour in the collection room. This result, together with the shortage of publications on prolactin response to stress in dogs [58], strongly suggests the need for future research, focused on acute stressors that might provide additional information on the possible use of prolactin as a biomarker of acute stress in domestic dogs. It must be noted that most dogs did not show fearful behaviours in the collection room. This could not be a consequence of their familiarity with this place, since visiting veterinary facilities occurred only for veterinary care, and surgical procedures. However, the presence of both familiar people (shelter staff) and familiar dogs (box mates) likely decreased the stress of novelty and blood withdraw in the collection room.

A possible explanation for cortisol results of the current study is that some dogs belonging to the sample could be chronically stressed and their cortisol levels mirroring such condition: in fact, sheltered dogs are frequently exposed to stressful conditions [87], and those of the current sample had experienced abandonment, abuse and/or neglect.

In other terms, the absence of correlation found between cortisol levels, stress score and fear behaviour in the collection room, as previously reported for the relationship between cortisol and prolactin levels, might be explained by the fact that cortisol values were affected by factors other than those analysed in this study. For instance, the long permanence in the shelter, lived by dogs in our sample, might have led to chronic stress, leading to the suppression and deregulation of the HPA axis [48]. In fact, several studies have reported cortisol variations depending on how long dogs have been in the shelter [88,89,90,91,92]. This point, together with other weaknesses previously reported for cortisol, make even more relevant the need for alternative easily measurable biomarkers of stress. 

It could be argued that, if prolactin levels remain constantly elevated during a long period of stress, then this hormone could be a good biomarker of chronic stress in the dog. If prolactin levels in long-stay sheltered dogs were elevated, while cortisol levels varied according to the welfare state of the dog, e.g., decreasing along with the permanence time in the shelter [90,91,92], then a negative correlation between prolactin and cortisol would be expected, as has been partially reported in the current study. It is advisable that further research investigate whether prolactin is a good candidate for being a biomarker of chronic stress in dogs. For this, it must be taken into account that, at the moment, the use of biological matrixes other than blood for measuring prolactin is not advisable in dogs [93].

This study presents some weaknesses and limitations. One limitation is that this study was focused on assessing the possible association between prolactin and other measures of stress, but no analysis was performed to investigate the possible impact of factors such as the number of dogs per box, the variability on previous individual experiences, etc. In addition, shared experiences such as living in the same cage might have an impact and lead to a cage effect, which was not investigated in the current research. Future studies, involving a wider sample, should take these factors into account. Another limitation is that, although on one hand the homogeneity of the sample may have reduced variability in the data, the specific characteristics of dogs (breed, sex, sexual status, previous experience, and management) require caution in generalizing results to the whole canine population, in particular to pet dogs, whose previous experiences might lead them to respond differently to stress. A potential weakness of this study is related to the lack of knowledge about the timing of prolactin release and decline after a stressful event, especially in the canine species. Future research should better investigate this point.

## 5. Conclusions

The study of the serum levels of cortisol and prolactin, and stress behaviour in dogs has not resulted in any positive correlation between variables, and the display of fearful behaviour at the moment of blood sampling seems to not have influenced the assessed parameters. Cortisol levels may depend not only on the observational factors scored in this study (stress score and fearful behaviour), but also on the possible presence of a chronic stress. This might explain the weak negative correlation found with prolactin values. This weak negative correlation, together with the high serum prolactin levels compared to those reported by other authors for healthy domestic dogs, may indicate that prolactin could be a good biomarker of chronic stress.

The evaluation of welfare in kennelled animals is essential to ensure and improve their quality of life, and the importance of a multiparametric approach has been stressed [48]. The study of behavioural and physiological parameters other than cortisol might be very helpful to increase the robustness of these studies and consequently provide a useful tool to facilitate adoptions, apply proper environmental enrichment improvements, or target dogs for behavioural or clinical intervention. 

## Figures and Tables

**Table 1:** Number of dogs and relative percentage (%, calculated on the total sample of 40 dogs) displaying each of the 18 stress-related behaviours, and number of occurrences (times the behaviour was displayed).

Behaviour	Number and % of Dogs Displaying the Behaviour	Number of Occurrences	Behaviour	Number and % of Dogs Displaying the Behaviour	Number of Occurrences
Tongue out	20; 50.0%	101	Circling	3; 7.5%	3
Panting	4; 10.0%	38	Eliminating	3; 7.5%	3
Yawning	15; 37.5%	34	Howling	2; 5.0%	3
Paw lifting	1; 2.5%	21	Turn head	2; 5.0%	2
Behaviour against the fence	3; 7.5%	12	Tuck tail	1; 2.5%	1
Shaking	9; 22.5%	10	Growling	0; 0.0%	0
Pacing	3; 7.5%	7	Hiding	0; 0.0%	0
Cowering	4; 10.0%	6	Salivation	0; 0.0%	0
Whining	4; 10.0%	5	Trembling	0; 0.0%	0
